# Type 1 Diabetic Neuropathy and C-peptide

**DOI:** 10.1080/15438600490424541

**Published:** 2004

**Authors:** Anders A. F. Sima, Weixian Zhang, George Grunberger

**Affiliations:** 1 Department of Pathology Wayne State University Detroit Michigan USA; 2 Department of Neurology Wayne State University Detroit Michigan USA; 3 The Morris Hood Comprehensive Diabetes Center Wayne State University Detroit Michigan USA; 4 Department of The Grunberger Diabetes Institute Bloomfield Hills Michigan USA; 5 Department of Pathology Scott Hall Room 9275 540 East Canfield Avenue, Detroit MI 48201 USA

## Abstract

The most common microvascular diabetic complication, diabetic peripheral polyneuropathy (DPN), affects type 1 diabetic patients more often and more severely. In recent decades, it has become increasingly clear that perpetuating pathogenetic mechanisms, molecular, functional, and structural changes and ultimately the clinical expression of DPN differ between the two major types of diabetes. Impaired insulin/C-peptide action has emerged as a crucial factor to account for the disproportionate burden affecting type 1 patients. C-peptide was long believed to be biologically inactive. However, it has now been shown to have a number of insulin-like glucoseindependent effects. Preclinical studies have demonstrated dose-dependent effects on Na^+^,K^+^-ATPase activity, endothelial nitric oxide synthase (eNOS), and endoneurial blood flow. Furthermore, it has regulatory effects on neurotrophic factors and molecules pivotal to the integrity of the nodal and paranodal apparatus and modulatory effects on apoptotic phenomena affecting the diabetic nervous system. In animal studies, C-peptide improves nerve conduction abnormalities, prevents nodal degenerative changes, characteristic of type 1 DPN, promotes nerve fiber regeneration, and prevents apoptosis of central and peripheral nerve cell constituents. Limited clinical trials have confirmed the beneficial effects of C-peptide on autonomic and somatic nerve function in patients with type 1 DPN. Therefore, evidence accumulates that replacement of C-peptide in type 1 diabetes prevents and even improves DPN. Large-scale food and drug administration (FDA)-approved clinical trials are necessary to make this natural substance available to the globally increasing type 1 diabetic population.


*Experimental Diab. Res.*, 5:65–77, 2004
Copyright © Taylor and Francis Inc.
ISSN: 1543-8600 print / 1543-8619 online
DOI: 10.1080/15438600490424541

### Type 1 Diabetic Neuropathy and C-peptide

**Anders A. F. Sima,[1,2,3] Weixian Zhang,[1,3] and George Grunberger[4]**

*Departments of [1]Pathology and [2]Neurology, and [3]The Morris Hood Comprehensive Diabetes Center, Wayne State University, Detroit, Michigan, USA*
*[4]The Grunberger Diabetes Institute, Bloomfield Hills, Michigan, USA*

**The most common microvascular diabetic complication, diabetic peripheral polyneuropathy (DPN), affects type 1 diabetic patients more often and more severely. In recent decades, it has become increasingly clear that perpetuating pathogenetic mechanisms, molecular, functional, and structural changes and ultimately the clinical expression of DPN differ between the two major types of diabetes. Impaired insulin/C-peptide action has emerged as a crucial factor to account for the disproportionate burden affecting type 1 patients. C-peptide was long believed to be biologically inactive. However, it has now been shown to have a number of insulin-like glucose-independent effects. Preclinical studies have demonstrated dose-dependent effects on $Na^+$,$K^+$-ATPase activity, endothelial nitric oxide synthase (eNOS), and endoneurial blood flow. Furthermore, it has regulatory effects on neurotrophic factors and molecules pivotal to the integrity of the nodal and paranodal apparatus and modulatory effects on apoptotic phenomena affecting the diabetic nervous system. In animal studies, C-peptide improves nerve conduction abnormalities, prevents nodal degenerative changes, characteristic of type 1 DPN, promotes nerve fiber regeneration, and prevents apoptosis of central and peripheral nerve cell constituents. Limited clinical trials have confirmed the beneficial effects of C-peptide on autonomic and somatic nerve function in patients with type 1 DPN. Therefore, evidence accumulates that replacement of C-peptide in type 1 diabetes prevents and even improves DPN. Large-scale food and drug administration (FDA)-approved clinical trials are necessary to make this natural substance available to the globally increasing type 1 diabetic population.**

**Keywords** Axonal Degeneration; C-peptide; Diabetic Neuropathy; Nodal Degeneration; Regeneration

### INTRODUCTION

Diabetic neuropathy is a group of disorders and as such the most common chronic diabetic complication and affects both type 1 and type 2 diabetic patients (Greene et al., 1997; Sima, 2003a). Despite decades of intensive clinical and experimental investigations, diabetic neuropathy remains illusive. Diabetic neuropathy includes several distinct syndromes, of which symmetric, mainly sensory polyneuropathy, often coupled with autonomic polyneuropathy, is the most common and is referred to as diabetic polyneuropathy (DPN).

The prevalence of DPN varies from 10% within a year of diagnosis to 50% in patients with diabetes for 25 years or longer (Pirart, 1977; Vinik et al., 1992; Sima, 1994), and with an average prevalence of 30% (Tesfaye et al., 1996). DPN accompanying type 1 diabetes occurs more predictably, progresses more rapidly, resulting in a more severe neuropathy (Dyck et al., 1999). Close to 100% of type 1 patients eventually develop DPN (Vinik et al., 1992). The underlying causes of DPN are multiple and involve genetic predispositions and several interrelated metabolic and molecular abnormalities consequent to hyperglycemia and insulin and C-peptide deficiencies (Greene et al., 1992, 1997; Sima, 1996, 2003a, 2003b; Forst et al., 1998a; Low et al., 1999; Sima and Sugimoto, 1999;

Received 2 August 2003; accepted 3 November 2003.
The authors wish to acknowledge numerous students, fellows, and colleagues who have contributed substantially to much of the work cited in this review. These studies were supported in part by grants from NIH, the Juvenile Diabetes Research Foundation, Thomas Foundation, American Diabetes Association, Canadian Diabetes Association, and the Medical Research Council of Canada.
Address correspondence to Anders A. F. Sima, MD, PhD, FRCP(C), Department of Pathology, Scott Hall, Room 9275, 540 East Canfield Avenue, Detroit, MI 48201, USA. E-mail: asima@med.wayne.edu

Sugimoto et al., 2000a). During the last three decades, several experimental drugs targeting specific mechanisms have undergone clinical testing. However, the results from these trials have been disappointing, which in part may be due to the fact that therapeutic interventions have occurred too late in the natural history of DPN or applied compounds have not shown required potency (Oats and Mylari, 1999; Sima, 2001).

Here we review the key pathogenetic factors involved in DPN. Differences in underlying mechanisms in type 1 and type 2 DPNs will be discussed. Clinical and experimental findings following C-peptide replacement will be dealt with with respect to metabolic abnormalities, nerve regeneration, nodal degeneration, and functional deficits.

### THE NATURAL HISTORY OF DPN IN TYPE 1 AND TYPE 2 DIABETES

Data concerning the early development of DPN have almost exclusively been obtained from animal models. Hyperglycemic streptozotocin (STZ)-induced diabetes in rats or spontaneously type 1 diabetic BB/Wor-rats show, within weeks of onset, significant decreases in motor and sensory nerve conduction velocities (NCVs), associated with increased activity of the polyol pathway, decreased endoneurial blood flow, and impaired neural $Na^+$,$K^+$-ATPase and NO activities (Sima and Sugimoto, 1999) (Figure 1). These early functional deficits are reversible at this "metabolic stage" of DPN. They are associated with reversible axonal swellings at the node of Ranvier, secondary to increased intra-axonal $Na^+$ due to the impaired $Na^+$,$K^+$-ATPase activity (Brismar and Sima, 1981; Sima and Brismar, 1985). Additional pathogenetic components are progressively emerging, such as oxidative stress and a progressive decline in the activities of neurotrophic factors, like nerve growth factor (NGF) and the insulin-like growth factor (IGF) system (Brewster et al., 1994; Sima and Sugimoto, 1999; Tomlinson and Fernyhough, 1999). Simultaneously, structural changes develop consisting of axonal atrophy and axonal dying-back degeneration, which occur in a length-dependent manner ("the structural phase"). These changes contribute to the progressively less reversible NCV defect (Brismar et al., 1987). Type 1 DPN in both humans and experimental animal models show additional structural abnormalities of the nodal and paranodal apparatus (Brismar et al., 1987; Sima et al., 1988b), changes that do not occur in type 2 DPN (Sima et al., 1986, 2000) (Figure 2). Experimentally, these changes are associated with lateralization of nodal $Na^+$ channel and result in a conduction block of affected fibers, hence contributing to the progressively less reversible and more severe NCV deficits in type 1 DPN (Brismar et al., 1987; Cherian et al., 1996; Sima et al., 2000). Progressive axonal degeneration, coupled with impaired regenerative capacity, in the type 1 diabetic BB/Wor rat result in a progressive nerve

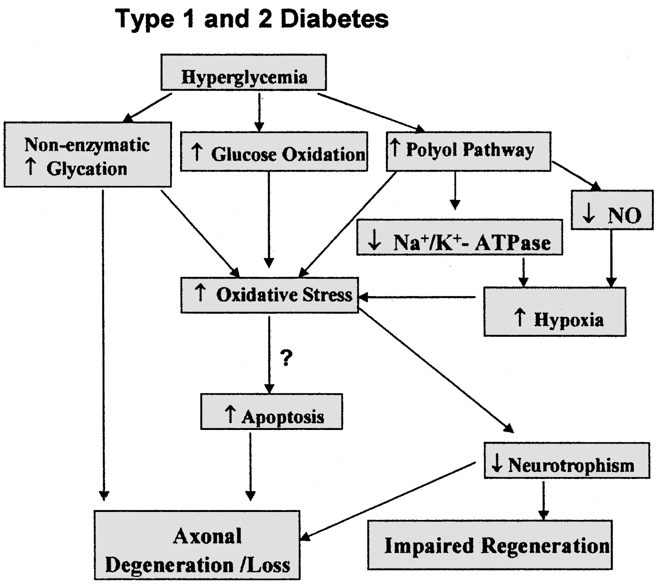

**FIGURE 1**
Pathogenetic mechanisms evolving from hyperglycemia common to type 1 and type 2 diabetes. Poloyl pathway activation perturbs $Na^+$,$K^+$-ATPase and NO activities causing the acute "metabolic" reversible nerve conduction defect. Several mechanisms contribute to oxidative stress, which in turn is believed to affect neurotrophic support mechanisms and possibly apoptosis. These abnormalities lead to axonal degeneration and loss as well as impaired nerve regeneration, which constitute the "structural phase" of DPN. During this phase, nerve conduction defects become increasingly irreversible. Note nodal/paranodal degeneration (cf., Figure 2) is not believed to be caused by hyperglycemia per se.

fiber loss, which is significantly milder in its type 2 counterpart, the BBZDR/Wor rat (Sima et al., 2000).

In human DPN, the spectrum of somatic DPN can be divided into reversible and persistent syndromes (Sima et al., 1997b; Thomas, 1997). The latter is classified into sensory and motor syndromes of increasing severity, which reflect the natural history of DPN. Part of the problem in staging and classifying DPN is that the progression rates of objective functional measurements are not linear and differ between nerves (Laudadio and Sima, 1996, 1998), reflecting the length-dependent axonal dying-back phenomenon.

Differences exist in the neuropathology of DPN in the two types of diabetes. The sequence of nodal and paranodal changes, consisting of axoglial dysjunction, paranodal demyelination, and intercalated internodes, is characteristic of type 1 human DPN, but does not occur in type 2 DPN (Sima et al., 1988b), hence showing the same differences as in experimental type 1 and type 2 DPNs. On the other hand, primary segmental demyelination tends to be more characteristic of type 2 DPN.

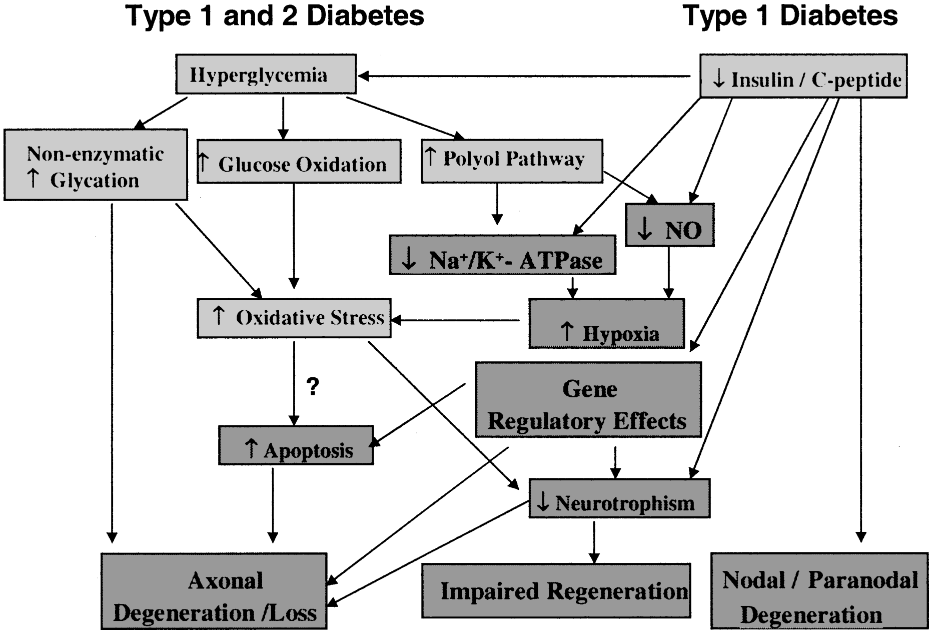

FIGURE 2

Scheme of pathogenetic mechanisms operable in type 1 DPN. These are initiated by hyperglycemia (cf., Figure 1) and additive effects of insulin/C-peptide deficiencies. C-peptide deficiency affects independently $Na^+$,$K^+$-ATPase and NO, causing hypoxia. Furthermore C-peptide deficiency appears to affect neurotrophic mechanisms through gene-regulatory mechanism. This appears to be more severe than that caused by oxidative stress, which is not effected by C-peptide treatment. Impaired neurotrophism results in axonal degeneration and loss and impaired regeneration. Insulin/C-peptide deficiencies appear to be exclusively responsible for the progressive nodal and paranodal degenerative changes. Therefore insulin/C-peptide deficiencies appears to several pathogenetic mechanisms also caused by hyperglycemia as well as initiate specific mechanisms.

These differences are likely to affect nerve function differently and are likely to account for the clinically more severe DPN in type 1 diabetic patients (Sima and Cherian, 1997; Dyck et al., 1999; Sugimoto et al., 2000a). Nevertheless, both type 1 and type 2 human DPNs shows progressive nerve fiber loss (Sima et al., 1988a). It is therefore becoming increasingly evident that fundamental differences do exist between DPN in type 1 versus type 2 diabetes.

### ESTABLISHED PATHOGENETIC MECHANISMS

The elucidation of the pathogenetic mechanisms underlying DPN has largely been performed in acutely STZ-diabetic rat. In doing so, no distinctions were made as to potential differences between DPN in the two major types of diabetes, because this diabetic model is a poor model of the human disorders. Instead the data from acutely STZ-diabetic rats were extrapolated to the chronic and diverse human diseases.

Nevertheless, several mechanisms have been revealed, some of which show a sustained action from the acute phase of DPN into the more chronic stage also in the human disorders. These include the activation of the polyol pathway, oxidative stress, nonenzymatic glycation, and impaired neurotrophic sustenance. Because these underlying mechanisms are, in part, fueled by hyperglycemia (Figure 1), it was and still is believed that the basic and only culprit in the development of the microvascular complications is hyperglycemia, which, hence conveniently, would explain the development of these complications in both type 1 and type 2 patients (Dyck and Dyck, 1999; Feldman, 2003).

### Polyol Pathway and Associated Abnormalities

The shunting of excessive glucose through the polyol pathway leads to accumulation of intracellular osmolytes, such as sorbitol and fructose, at the expense of others, such as *myo*-inositol and taurin (Aruoma et al., 1988; Stevens et al., 1993; Greene et al., 1993). This has several consequences. Depletion of *myo*-inositol results in impaired diacylglycerol to maintain protein kinase C, necessary for activation of $Na^+$,$K^+$-ATPase (Green et al., 1987; Zhu and Eichberg, 1990; Ishii et al., 1998). Depletion of taurin through activation of the polyol pathway impairs its action as an endogenous antioxidant and neurotrophic factor (Aruoma et al., 1988; Stevens et al., 1993; Greene et al.,

1993). Furthermore, activation of the polyol pathway depletes NADPH, a cofactor of aldose reductase, glutathione reductase, and endothelial nitric oxide (NO) synthase (eNOS), hence resulting in a decrease in reduced glutathione and impaired NO formation (Figure 1) (Greene et al., 1993; Stevens et al., 1994). The former results in decreased defense capabilities against oxidative stress and the latter promotes vascular constriction and impaired nutritive blood flow. To these biochemical aberrations should be added that polymorphisms of the initiation site of the aldose reductase gene in type 1 diabetic patients are associated with higher frequencies of DPN and diabetic nephropathy (Heesom et al., 1998; Oates and Mylari, 1999). It is therefore clear that activation of the polyol pathway has a pathogenetic role to play and that it promotes other mechanisms indirectly, such as oxidative stress, impaired nutritive blood flow, and neurotrophic support (Figure 1).

Numerous aldose reductase inhibitor trials were performed in the 1980s and 1990s with disappointing results. The reasons for this may be several: (1) the underlying rationale was obtained from data from short-term prevention studies mainly in STZ-induced diabetic rats; (2) the interventions in human DPN was too late in its natural history, and (3) many of the compounds were not potent enough to achieve an efficacious inhibition of the enzyme. In retrospect, the failures with aldose reductase inhibitors underpins the danger of extrapolating acute animal data to a much more complex chronic but highly dynamic human disease process (Sima, 2003a, 2003b).

### Nonenzymatic Glycation and Oxidative Stress

The glycation process is enhanced in diabetic nerve in humans and animal models (Vlassara et al., 1981; Araki et al., 1992; Soulis et al., 1997; Hammes et al., 1999) (Figure 1). Glycation of neuronal cytoskeletal proteins, such as neurofilaments, tubulin, and actin, is likely to contribute to slowing of axonal transport, atrophy, and degeneration (Cullum et al., 1991; Ryle et al., 1997) (Figure 1). Glycation of laminin, a key constituent of Schwann cell basal lamina, which is important in nerve sprouting, may contribute to impaired nerve fiber regeneration, characteristic of DPN (Federoff et al., 1993). Several myelin proteins, such as p-zero (PO), myelin basic protein, and proteolipid protein, are glycated in diabetes and are recognized by scavenging macrophages via receptor for advanced glycationend product (RAGE) (Vlassara et al., 1984, 1985) and may hence play a contributing role in segmental demyelination.

Oxidative stress facilitates the formation of glycoxidation products such as carboxymethyllsine (CML) and pentosidine (Baynes, 1991). There are several potential sources of oxidative stress in diabetes, including altered redox status (Williamson et al., 1993), dysregulation of glutathione synthesis (Yoshida et al., 1995), and hypoxia and ischemic reperfusion injury (McCord, 1985) (Figure 1). Glucose autoxidation and glycoxidation, which are catalyzed by trace amounts of transition metal ions, generate reactive oxygen species (Hunt and Wolff, 1991; Hunt et al., 1988) (Figure 1). Low-dose transition metal chelators, such as deferoxamine and trientine, improve nerve blood flow and NCV in the STZ-diabetic rat (Cameron and Cotter, 1995). Oxidative stress gives rise to breakdown of endothelial barrier functions and nuclear factor (NF)-$\kappa$B–mediated gene inductions of tissue factor and endothelin-1, both of which contribute to reduced vascular blood flow (Bierhaus et al., 1997; Wautier et al., 1996). Tissue levels of glycoxidation products correlate with the severity of nephropathy, retinopathy, and vasculopathy in diabetic patients (Sell et al., 1992; Beisswenger et al., 1993; McCance et al., 1993).

$\alpha$-Lipoic acid is one of the most powerful antioxidants. A review of several clinical trials employing $\alpha$-lipoic acid showed reduction in neuropathic symptoms and small improvements of autonomic function (Ziegler et al., 1999). The likelihood of antioxidant therapy to be successful will probably require multiple antioxidant compounds targeting the different mechanisms and would include $\alpha$-lipoic acid, vitamins E and C, and probably agents targeting the perturbed lipid metabolism such as $\gamma$-linolenic acid (GLA), evening primrose oil/fish liver oil, and acetyl-L-carnitine (Cameron and Cotter, 1995; Sima and Sugimoto, 1999; Sima, 2001).

### Perturbed Neurotropism

Evidence indicates that impaired neurotrophic support is involved in diabetes-related neuronal dysfunctions (Figure 1) (Brewster et al., 1994; Tomlinson and Fernyhough, 1999). NGF is trophic to small-fiber sensory and sympathetic ganglion neurons (Ebendal, 1992). In the STZ-diabetic rat, reduced expression of NGF mRNA in muscle and skin (Brewster et al., 1994) and its impaired retrograde axonal transport (Jakobsen et al., 1981; Hellweg and Hartung, 1990) lead to impaired neurotrophic support of NGF-dependent neurons. NGF administration prevents the reduction of neuropeptides such as substance P and calcitonin gene-related peptide in dorsal root ganglion (DRG) neurons and sciatic nerve in diabetic rats (Apfel et al., 1994; Diemel et al., 1994). These neuropeptides are confined to small-fiber sensory neurons, mediating nociceptive, or thermoreceptive sensation (New and Mudge, 1986).

Neurotrophin-3 (NT-3), trophic for sympathetic neurons and sensory neurons of large-diameter fibers (Hory-Lee et al., 1993; Ernfors et al., 1994), is reduced in diabetic muscle. Administration of NT-3 ameliorates sensory NCV deficits, but not those of motor nerves (Tomlinson et al., 1997). Reduced expressions of the high-affinity receptors in respective neurons (Tomlinson et al., 1997; Fernyhough et al., 1998) and decreased synthesis of neurotrophins contribute to nerve dysfunction in DPN. Recent clinical trials have, however, been inconclusive with respect to the effect of recombinant human NGF (Apfel, 1999).

IGFs are neurotrophic to sensory, sympathetic, and motor neurons alike (Ishii, 1995). Reduction in systemic IGF-I levels and increased IGF-I–binding proteins contribute to impaired IGF-I activity in type 1 diabetic patients (Crosby et al., 1992). In the STZ-diabetic rat, the IGF-I mRNA expression is dramatically reduced in the liver and in the spinal cord, which correlates with significantly decreased conduction velocities in both spinal cord and peripheral nerves (Ishii et al., 1994; Wuarin et al., 1994). Both IGF-I and IGF-II mRNA expressions are significantly decreased in sciatic nerve of STZ diabetes (Wuarin et al., 1994) and well established in both peripheral nerve and the brain in the type 1 BB/Wor rat after 8 weeks of diabetes (Xu et al., 2002; Li et al., 2002a; Pierson et al., 2002, 2003a). Subcutaneous infusion of IGF-I or IGF-II prevents the progression of hyperalgesia in the STZ-diabetic rat (Zhuang et al., 1996), and local administration of IGFs protects against impairments of sensory nerve regeneration (Ishii and Lupien, 1995).

### THE ROLES OF INSULIN AND C-PEPTIDE

Lately, it has become clear that hyperglycemia, although an important etiological factor, is not the sole culprit in the development of diabetic complications. Increasing attention is being paid to insulin and/or C-peptide deficiencies. Both insulin and C-peptide exerts a number of metabolic, neuroprotective, and antiapoptotic effects (Sima et al., 2001a; Li et al., 2002b, 2003; Pierson et al., 2003b).

Proinsulin C-peptide enhances the effects of insulin (Johansson et al., 1993; Jensen and Messina, 1999; Grunberger et al., 2001; Li et al., 2001) and augments phosphorylation of the insulin receptor (Li et al., 2001, 2003; Grunberger et al., 2001). C-peptide signals through the insulin signaling pathway and stimulates glycogen synthesis and amino acid uptake by itself and enhances these effects by insulin within a narrow concentration range (Grunberger et al., 2001; Li et al., 2003; Grunberger and Sima, 2004) (see below). It was, therefore, suggested that C-peptide interacts with the insulin receptor, although the binding of C-peptide to a specific membrane receptor has also been suggested (Rigler et al., 1999).

Therefore, the pathophysiological role of proinsulin C-peptide deficiency has received increasing attention, with particular focus on the potential therapeutic value of C-peptide replacement in preventing and ameliorating type 1 diabetic complications.

### Metabolic Effects of C-peptide

C-peptide elicits concentration-dependent stimulation of $Na^+,K^+$-ATPase activity in a variety of tissues, including renal tubular cells, rat sciatic nerve, pancreatic islets, granulation tissue, and red blood cells (Figure 2) (Forst et al., 2000; Wahren et al., 2000; Zhang et al., 2001; Sima et al., 2001b). Further support for C-peptide's effects on $Na^+,K^+$-ATPase is provided by its effect on rat sciatic nerve $Na^+,K^+$-ATPase in type 1 diabetic BB/Wor rats treated with C-peptide for 8 months, an effect that is dose dependent (Sima et al., 2001b; Zhang et al., 2001). This is substantiated by partial correction of the associated defect in NCV and paranodal swelling, secondary to axonal $Na^+$ accumulation. Furthermore, in the C-peptide–deficient diabetic BB/Wor rat model, the expression of both insulin receptor and IGF-I receptor mRNA and protein in peripheral nerve and brain tissue are normalized by C-peptide replacement and the diabetes-induced hippocampal apoptosis is partially prevented by C-peptide replacement (Li et al., 2003).

Several studies demonstrate an effect of C-peptide on NO release. It stimulates eNOS with release of NO from bovine aortic endothelial cells in a concentration-dependent manner, an effect that is abolished by NOS inhibitors (Figure 2) (Kunt et al., 2003). This is in keeping with the finding that C-peptide induces increased forearm and skin blood flow in type 1 diabetic patients, which is blocked by a NOS blocker (Johansson et al., 1992; Forst et al., 1998a, 1998b; Forst and Kunt, 2004). It is also consistent with the demonstration of a C-peptide concentration-dependent dilatation of rat skeletal muscle arterioles in the presence of insulin (Menegoz et al., 1997). In the BB/Wor rat, C-peptide prevents the decrease in endoneurial blood flow but has no effect on oxidative stress (Sima et al., 2003a). The effect of C-peptide on endoneurial blood flow was also demonstrated in the STZ-diabetic rat (Cotter et al., 2003).

C-peptide has no effect on the polyol pathway activity; hence the effects on neural $Na^+,K^+$-ATPase and endothelial NO occur independently of this pathway (Figure 2). These data may account for a different distribution of the $Na^+,K^+$-ATPase defect in type 1 BB/Wor rats (C-peptide deficient) versus the type 2 BBZDR/Wor rat, in which the defect appears to affect mainly endothelial $Na^+,K^+$-ATPase (Sima et al., 2000). Furthermore, the independent correction of endothelial NO by C-peptide certainly contributes to normalization of endoneurial blood flow without invoking oxidative stress. Taken together, these beneficial metabolic effects are likely to account for the significant corrections of the acute nerve conduction defects, despite the presence of significant hyperglycemia. Oxidative stress, it has been suggested, may provide the final common pathway of the interactive metabolic abnormalities underlying microvascular complications (Feldman, 2003; Brownlee, 2001). However, the data cited above appear to challenge this concept, because

C-peptide administration corrects several metabolic abnormalities, which supposedly contribute to oxidative stress, as well as nerve function, although it does not affect oxidative stress per se.

### The Effect of C-peptide on Nerve Function

As would be expected from the corrective effects on neural $Na^+$,$K^+$ -ATPase, endothelial NO, and endoneurial blood flow, C-peptide significantly prevents both motor and sensory NCVs as well as thermal hyperalgesia, a function of C-fibers (Sima et al., 2001b, 2003b; Stevens et al., 2003). However, these effects are not complete but are associated with sometimes significant residual functional defects, which are most likely accounted for by additive hyperglycemic effects on underlying metabolic abnormalities (Figure 3). Apart from the acute effects on nerve functions in type 1 diabetic BB/Wor rats, sustained C-peptide replacement prevents significantly the chronic functional abnormalities (Sima et al., 2001b). These effects are associated with preventive effects on emerging structural abnormalities, particularly axonal degeneration and loss and probably most importantly the preventive effects on the specific type 1 degenerative changes affecting the nodal and paranodal apparatus (Sima et al., 2001b) (Figure 2) (see below). From a clinical viewpoint, a more encouraging finding is the therapeutic effect of C-peptide replacement on established experimental DPN, with significant functional improvements. These are associated and correlated with reparative effects on underlying structural abnormalities (Sima et al., 2001b) (see below). These findings suggest that insulinomimetic C-peptide has a ubiquitous effect on both myelinated and unmyelinated fiber populations and parallel a recent randomized, double-blind, placebo-controlled study, in which Ekberg and colleagues (2003) demonstrated that replacement with C-peptide (600 nmol/day) resulted in significant improvements in nerve function. C-peptide–treated patients showed significant (80%) improvement in sural NCV and vibration perception over a 3-month treatment period, as compared to patients treated with insulin alone. Earlier studies by the same group (Ekberg et al., 2003) also demonstrated beneficial effects on cardiac autonomic function as well as improved temperature threshold discrimination after C-peptide replacement of type 1 diabetic patients (Johansson et al., 2000). None of these effects were demonstrated in patients who received insulin therapy alone.

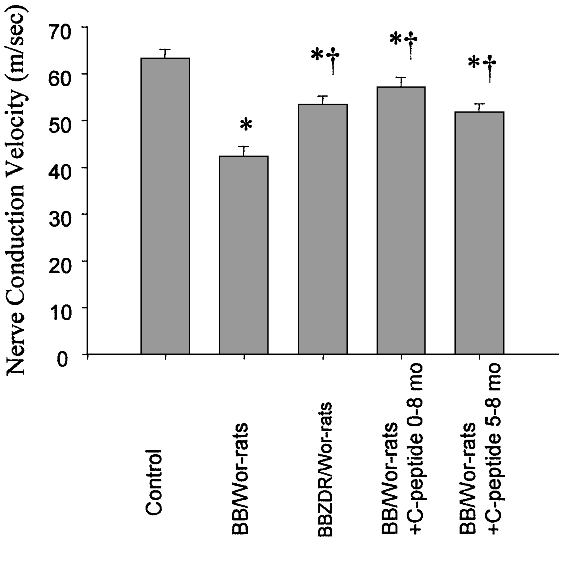

**FIGURE 3**

Nerve conduction velocities in age-matched control rats, 8-month type 1 BB/Wor and type 2 BBZDR/Wor-rats. Note a significantly ($P < .001$) nerve conduction defect in type 2 rats. Prevention with C-peptide (0 to 8 months) resulted in a significant ($P < .001$) amelioration of the conduction defect. Intervention with C-peptide (5 to 8 months) resulted in a significant ($P < .006$) improvement of nerve conduction. Note the nerve conductions in C-peptide–replaced animals were similar to those in type 2 diabetic rats, suggesting a hyperglycemic component and an insulin/C-peptide deficiency–related component. $^*P < .001$ versus control rats; $^\dagger P < .001$ versus BB/Wor rats (Data from Sima et al., 2001).

### Effect of C-peptide on Axonal Degeneration and Loss

Long-term preventive studies in the spontaneously type 1 diabetic BB/Wor rat have demonstrated significant preventive effects on axonal atrophy, degeneration, and loss of myelinated fibers (Sima et al., 2001b, 2002, 2003b) (Figures 4 and 5). Similarly, C-peptide prevents atrophy and loss of unmyelinated fibers in the sural nerve of the same model (Sima and Murakawa, unpublished data). However, these effects on myelinated and unmyelinated fiber pathology was not always complete but left a significantly milder residual DPN, which was not significantly different from the markedly milder neuropathy caused by hyperglycemic and hyperinsulinemic type 2 diabetes in BBZDR/Wor rats (Sima et al., 2001b). Corresponding to the interventional effects on nerve function described above, intervention with C-peptide from 5 to 8 months of diabetes resulted in an 80% improvement in axonal degenerative changes and significant protections against myelinated and unmyelinated fiber loss (Sima et al., 2001b; Sima and Murakawa, unpublished data). Both the preventive and therapeutic effects of C-peptide are likely to be mediated by the corrective effects on the expression of neurotrophic factors transcending to normalization of the expression of key neuroskeletal proteins such as $\beta$-tubulins and neurofilaments (see below).

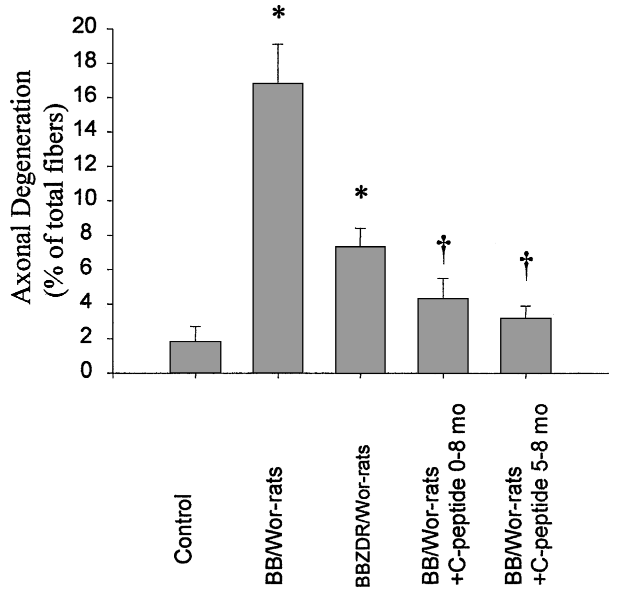

**FIGURE 4**

Frequency of myelinated fibers of the sural nerve showing axonal degeneration in the same groups of animals as in Figure 3. BB/Wor rats showed a 10-fold increase in axonal degeneration ($P < .001$) and a 4-fold ($P < .001$) increase in type 1 BBZDR/Wor rats. Both C-peptide prevention and intervention reduced the frequencies of axonal degeneration to approximately 2-fold of control rats, which were significant compared to untreated BB/Wor rats ($P < .001$). $^{*}P < .001$ versus control rats; $^{\dagger}P < .001$ versus untreated BB/Wor rats.

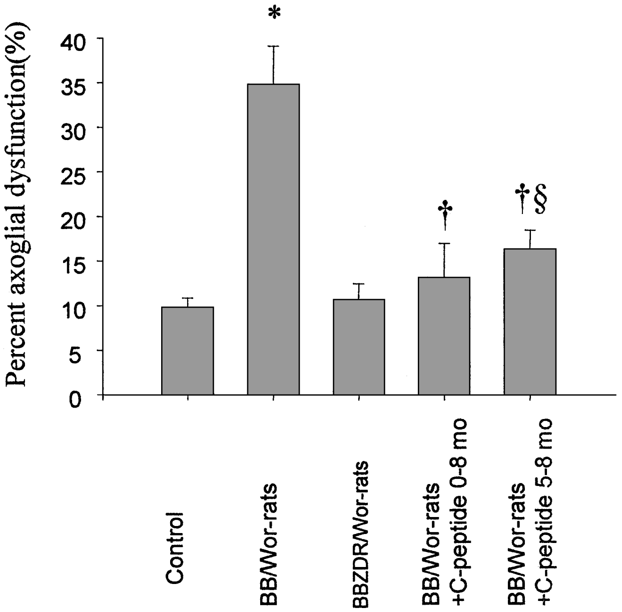

**FIGURE 5**

Frequencies of axoglial dysjunction, the hallmark of type 1 DPN, assessed by ultrastructural morphometry. Animal groups are the same as in Figure 3. Axoglial dysjunction was increased 3.5-fold ($P < .001$) compared to control rats, whereas no significant axoglial dysjunction occurred in type 2 BBZDR/Wor rats. C-peptide prevention and intervention resulted in significant ($P < .001$) decreases in the frequencies of axoglial dysjunction, although in the intervention group (5 to 8 months), a residual ($P < .05$) defect remained compared to control rats. Data are obtained from Sima et al., 2001. $^{*}P < .001$ versus control rats; $^{\dagger}P < .001$ vs nontreated BB/Wor rats; $^{\S}P < .05$ versus control rats.

### Effect of C-peptide on Neurotrophism

Evidence indicates that impaired neurotrophic support plays a role in DPN (Brewster et al., 1994; Tomlinson and Fernyhough, 1999). NGF is selectively trophic to small fiber sensory and sympathetic ganglia (Ebendal, 1992). Reduced expression of NGF and its impaired retrograde axonal transport lead to impaired trophic support of NGF-dependent neurons (Jakobsen et al., 1981; Hellweg and Harting, 1990). Furthermore, impairments in NGF receptors are likely to contribute to reduced responses. The low affinity p75 receptor undergoes increased turnover in diabetic nerve (Hruska et al., 1993). Expression of the high-affinity TrkA receptor is markedly reduced in DRG of BB/Wor rats but not in type 2 BBZDR/Wor rats (Pierson et al., 2002). Replenishment of C-peptide in the type 1 BB/Wor rat totally prevents the defect in TrkA receptor expression (Pierson et al., 2003b).

IGFs have neurotrophic actions on sensory, sympathetic, and motor neurons (Ishii, 1995). In the STZ-diabetic rat and BB/Wor rat, IGF-I mRNA expression is dramatically reduced in peripheral nerve, DRG, and spinal cord (Ishii et al., 1994; Wuarin et al., 1994; Xu et al., 2002). Interestingly, in the BB/Wor rat, the IGF-I receptor is up-regulated in peripheral nerve and DRG but down-regulated in the brain (Pierson et al., 2003a, 2003b; Xu et al., 2002; Li et al., 2002a). These abnormalities are significantly milder in the spontaneously type 2 diabetic BB/Z rat and unaltered in the fa/fa rat (Zhuang et al., 1997; Pierson et al., 2002, 2003a). In the STZ-diabetic rat, subcutaneous infusion of IGF-I or IGF-II prevents the progression of hyperalgesia (Zhuang et al., 1996) and local administration of IGFs protects against impairments in sensory nerve regeneration (Ishii and Lupien, 1995). The abnormalities in IGF-I and IGF-I receptor expression in peripheral nerve, DRG, and brain in the type 1 BB/Wor rat are normalized by C-peptide replacement (Pierson et al., 2003b; Li et al., 2002a), believed to be related to a phosphoinositol 3-kinase (PI3K)-mediated effect on NF-$\kappa$B (Li et al., 2003).

### Effect of C-peptide on Nerve Regeneration

Progressive nerve fiber loss in DPN (Thomas and Eliasson, 1984; Greene et al., 1997) is in part due to impaired nerve fiber regeneration (Sima et al., 1988a). Nerve regeneration is a tightly regulated spatiotemporal sequence of events involving immediate early gene responses such as IGF-I → c-fos → NGF (Hengerer et al., 1990; Xu and Sima, 2001), activation of interleukins and cytokines, macrophage recruitment, wallerian degeneration, induction of cytoskeletal protein synthesis, and finally axonal sprouting, elongation, and maturation (Ide, 1996). Several of these components are perturbed in type 1 diabetes, such as altered immediate early gene responses (Xu and Sima, 2001; Pierson et al., 2002, 2003a), delayed wallerian degeneration, delayed onset and rate of regeneration, and impaired maturation of regenerated fibers (Kamijo et al., 1996). Cytoskeletal proteins synthesized in DRG are affected by DPN. Both perturbed synthesis and slowed transport of neurofilaments have been described. The importance of neurofilaments in determining axonal caliber and, thereby, conduction velocity has been demonstrated (Yagihashi et al., 1990; Ide, 1996; Kamijo et al., 1996; Sima, 1999). Tubulins are major components of microtubules. Together with actin filaments, microtubules play important roles in directional outgrowth of neurites, such as growth cone advance and polarity (Mitchison and Kirschner, 1988). Alterations in microtubule proteins affecting the assembly and stability of microtubules are therefore likely to modify axonal function and to influence neuronal remodeling and regeneration.

Significantly delayed and suppressed immediate early gene responses, impaired macrophage recruitment, and wallerian degeneration have been demonstrated in the type 1 BB/Wor rat (Kamijo et al., 1996; Sima et al., 1997a; Xu and Sima, 2001; Xu et al., 2002; Pierson et al., 2002, 2003b). These abnormalities are associated with impaired up-regulation of tubulin and a lack of normal down-regulation of neurofilament expression in DRGs preceded by a down-regulation of IGF-I, trkA, and p75 in DRG ganglion cells (Xu et al., 2002). This led us to suggest that tubulin expression exerts a negative feedback on neurofilament expression to facilitate the early transport of tubulins to initiate the growth cone extension (Xu et al., 2002). The sequence of abnormalities ultimately resulted in impaired axonal caliber growth and extension. Parallel studies in the isohyperglycemic and hyperinsulinemic type 2 BB/Z rat failed to demonstrate any major abnormalities in this sequence of events and showed more robust nerve fiber regeneration (Pierson et al., 2003a). C-peptide–replaced type 1 rats exhibited mild changes compared to nonreplaced animals (Pierson et al., 2003b). C-peptide normalized the immediate early gene response and the expression of neurotrophic factors and their receptors, tubulin, and neurofilaments in DRG neurons, resulting in normalization of axonal caliber growth and improvement of the elongation of regenerating fibers. These findings are likely to contribute to the prevention of nerve fiber loss in type 1 BB/Wor rats replenished with C-peptide (Sima et al., 2001b) and suggest that impaired nerve regeneration is a more prominent phenomenon in type 1 DPN and may contribute to the more severe clinical expression of DPN in this type of diabetes. Impaired nerve regeneration appears to be mainly the result of impaired insulin/C-peptide action rather than hyperglycemia.

### Effect of C-peptide on Nodal and Paranodal Changes

The high-affinity insulin receptor in peripheral nerve is localized to the nodal axolemma and to paranodal tight junctions (Sugimoto et al., 2000b). The protein expression of the insulin receptor is compensatorily overexpressed in peripheral nerve of BB/Wor rats, which is corrected by C-peptide, and underexpressed in the hyperinsulinemic type 2 BBZDR/Wor rat (Pierson et al., 2002, 2003b).

The molecular components of the node of Ranvier and the paranodal apparatus and their interactive regulation are complex and not fully understood. Voltage-gated $Na^+$ channels are located to the nodal apparatus and are responsible for action potential initiation and conduction. They consist of a pore-forming $\alpha$-subunit and two auxiliary subunits: $\beta_1$ and $\beta_2$. Cytoskeletal interactions with spectrin, actin, and contactin (Scherer, 1996; Lambert et al., 1997) occur through ankyrin$_G$ and appear to be critical to $Na^+$ channel and $Na^+$,$K^+$-ATPase enrichment at the node (Muller-Husman et al., 1993; Malhotra et al., 2000; Isom, 2002). The $\beta_1$ subunit, but not that of $\beta_2$, interacts with receptor tyrosine phosphatase $\beta$ (RTPT$_\beta$), which in turn acts as a ligand for the neuroreceptor contactin (Peles et al., 1995; Ratcliff et al., 2000). RTPT$_\beta$ is part of the insulin and NGF signaling pathways.

At the paranode, caspr makes up the tight junctions. These are also associated with spectrin, actin, and contactin, which, via $\beta_1$ $Na^+$ channel subunit and RTPT$_\beta$, interact with caspr (Einheber et al., 1997; Menegoz et al., 1997; Peles et al., 1997). SH3 domains of caspr appear to be responsible for the protein-protein interaction and bind with p85, the regulatory subunit of PI3K. Type 1 diabetic BB/W rat show a down-regulation of several key nodal and paranodal molecules, such as contactin, $\beta_1$ $Na^+$ channel subunit, caspr, and RTPT$_\beta$ (Sima et al., 2003a). This does not occur in the type 2 BB/Z rat and is prevented by replenishing C-peptide in type 1 diabetic rats. Interestingly the expression of the pore-forming $\alpha$ $Na^+$ channel subunit is not altered in either type 1 or type 2 BB rats. Post-translational modifications of paranodal and nodal molecules examined in human neuroblastoma cells SH-SY5Y show that maximal p85 binding to caspr and serin phosphorylation of ankyrin$_G$ occur only in the presence of both insulin and C-peptide. These modifications are necessary for protein-protein interaction at the paranode and the node, respectively (Sima et al., 2003b). Hence these data indicate that insulin and C-peptide deficiencies perturb the expression of crucial nodal and paranodal molecules and that impaired insulin action is likely to interfere with their assembly, thereby leading to the progressive disruption of the paranodal apparatus, which characterizes type 1 DPN in humans and rodents alike. This is further supported by the intimate colocalization of the insulin-receptor with paranodal tight junctions and the nodal membrane (Sugimoto et al., 2000b). These data therefore indicate that insulin/C-peptide deficiencies are mainly responsible for the progressive nodal and paranodal degenerative changes, which set the more severe type 1 DPN apart from its type 2 counterpart in both human and experimental rodent models.

### CONCLUDING THOUGHTS

By utilizing animal models that closely mimic the two major types of human diabetes, it is becoming increasingly evident that fundamental differences exist with respect to underlying pathogenetic mechanisms of the DPN occurring in type 1 versus type 2 diabetes. The most evident difference between the two types of diabetes is the presence and absence of insulin/C-peptide action. As reviewed in this article, the insulinomimetic effects of C-peptide is capable of preventing and in some instances improving established metabolic and functional and structural abnormalities, ameliorating aberrations in neurotrophic support and dysregulation of a series of interactive nodal and paranodal molecules. This is not to say that hyperglycemia is not an important player in the development of DPN, but we would argue that impaired insulin/C-peptide actions appears to be at least an equally important offender in the evolution of type 1 DPN. Preclinical data and small clinical trials clearly indicate that substantial benefits can be gained from replacement of not only insulin (for blood glucose control), but also C-peptide in controlling type 1 diabetic DPN and probably other type 1 microvascular complications.

### REFERENCES

Apfel, S. C. (1999) Neurotrophic factors in the therapy of diabetic neuropathy. *Am. J. Med.,* **107,** 34S–42S.

Apfel, S. C., Arezzo, J. C., Brownlee, M., Federoff, H., and Kessler, J. A. (1994) Nerve growth factor administration protects against experimental diabetic sensory neuropathy. *Brain Res.,* **634,** 7–12.

Araki, N., Ueno, N., Chakrabarti, B., Morino, Y., and Horiuchi, S. (1992) Immunochemical evidence for the presence of advanced glycation end products in human lens proteins and its positive correlation with aging. *J. Biol. Chem.,* **267,** 10211–10214.

Aruoma, O. I., Halliwell, B., Hoey, B. M., and Butler, J. (1988) The antioxidant action of taurine, hypotaurine and their metabolic precursors. *Biochem. J.,* **256,** 251–255.

Baynes, J. W. (1991) Role of oxidative stress in development of complications in diabetes. *Diabetes,* **40,** 405–412.

Beisswenger, P. J., Moore, L. L., Brinck-Johnsen, T., and Curphey, T. J. (1993) Increased collagen-linked pentosidine levels and advanced glycosylation end products in early diabetic nephropathy. *J. Clin. Invest.,* **92,** 2212–2217.

Bierhaus, A., Chevion, S., Chevion, M., Hofmann, M., Quehenberger, P., Illmer, T., et al. (1997) Advanced glycation end product-induced activation of NF-$\kappa$B is suppressed by a-lipoic acid in cultured endothelial cells. *Diabetes,* **46,** 1481–1490.

Brewster, W. J., Fernyhough, P., Diemel, L. T., Mohiuddin, L., and Tomlinson, D. R. (1994) Diabetic neuropathy, nerve growth factor and other neurotrophic factors. *Trends Neurosci.,* **17,** 321–325.

Brismar, T., and Sima, A. A. F. (1981) Changes in nodal function in nerve fibres of the spontaneously diabetic BB-Wistar rat. Potential clamp analysis. *Acta Physiol. Scand.,* **113,** 499–506.

Brismar, T., Sima, A. A. F., and Greene, D. A. (1987) Reversible and irreversible nodal dysfunction in diabetic neuropathy. *Ann. Neurol.,* **21,** 504–507.

Brownlee, M. (2001) Biochemistry and molecular cell biology of diabetic complications. *Nature,* **414,** 813–820.

Cameron, N. E., and Cotter, M. A. (1995) Neurovascular dysfunction in diabetic rats. Potential contribution of autoxidation and free radicals examined using transition metal chelating agents. *J. Clin. Invest.,* **96,** 1159–1163.

Cotter, M., Ekberg, K., Wahren, J., and Cameron, N. E. (2003) Effects of proinsulin C-peptide in experimental diabetic neuropathy. *Diabetes,* **52,** 1812–1817.

Cherian, P. V., Kamijo, M., Angelides, K. J., and Sima, A. A. F. (1996) Nodal $Na^+$-channel displacement is associated with nerve conduction slowing in the chronically diabetic BB/W-rat. Prevention by an aldose reductase inhibitor. *J. Diabetes Complication,* **10,** 192–200.

Crosby, S. R., Tsigos, C., Anderton, C. D., Gordon, C., Young, R. J., and White, A. (1992) Elevated plasma insulin-like growth factor binding protein-1 levels in type 1 (insulin-dependent) diabetic patients with peripheral neuropathy. *Diabetologia,* **35,** 868–872.

Cullum, N. A., Mahon, J., Stringer, K., and McLean, W. G. (1991) Glycation of rat sciatic nerve tubulin in experimental diabetic mellitus. *Diabetologia,* **34,** 387–389.

Diemel, L. T., Brewster, W. J., Fernyhough, P., and Tomlinson, D. R. (1994) Expression of neuropeptides in experimental diabetes; effects of treatment with nerve growth factor or brain-derived neurotrophic factor. *Brain Res. Mol. Brain. Res.,* **21,** 171–175.

Dyck, P. J., Davies, J. L., Wilson, D. M., Service, F. J., Melton, L. J. III, and O'Brien, P. C. (1999) Risk factors for severity of diabetic polyneuropathy: Intensive longitudinal assessment of the Rochester Diabetic Neuropathy Study Cohort. *Diabetes Care,* **22,** 1479–1486.

Dyck, P. J. B., and Dyck, P. J. (1999) Diabetic polyneuropathy. In: *Diabetic Neuropathy,* 2nd ed., Edited by Dyck, P. J., and Thomas, P. K., pp. 255–278. Philadelphia, W.B. Saunders.

Ebendal, T. (1992) Function and evolution in the NGF family and its receptors. *J. Neurosci. Res.,* **32,** 461–470.

Ekberg, K., Brismar, T., Johansson, B.-L., Jonsson, B., Lindström, P., and Wahren, J. (2003) Amelioration of sensory nerve dysfunction by C-peptide in patients with type 1 diabetes. *Diabetes,* **52,** 536–541.

Einheber, S., Zanazzi, G., Ching, W., Scheres, S., Milner, T. A., Peles, E., et al. (1997) The axonal membrane Caspr, a homologue of neurexin IV, is a component of the septate-like paranodal junctions that assemble during myelination. *J. Cell Biol.,* **139,** 1495–1506.

Ernfors, P., Lee, K. F., Kucera, J., and Jaenisch, R. (1994) Lack of neurotrophin-3 leads to deficiencies in the peripheral nervous system and loss of limb proprioceptive afferents. *Cell,* **77,** 503–512.

Federoff, H. J., Lawrence, D., and Brownlee, M. (1993) Nonenzymatic glycosylation of laminin and laminin peptide CIKVAVS inhibits neurite outgrowth. *Diabetes,* **42,** 509–513.

Feldman, E. L. (2003) Oxidative stress and diabetic neuropathy: A new understanding of an old problem. *J. Clin. Invest.,* **111,** 431–433.

Fernyhough, P., Diemel, L. T., and Tomlinson, D. R. (1998) Target tissue production and axonal transport of neurotrophin-3 are reduced in streptozotocin-diabetic rats. *Diabetologia,* **41,** 300–306.

Forst, T., DeLaTour, D. D., Kunt, T., Pfützner, A., Goitom, K., Pohlmann, T., et al. (2000) Effects of proinsulin C-peptide on nitric oxide, microvascular blood flow and erythrocyte $Na^+$,$K^+$ ATPase activity in diabetes mellitus type 1. *Clin. Sci.,* **98,** 283–290.

Forst, T., and Kunt, T. (2004) Effects of C-peptide on microvascular blood flow and blood haemorheology. *J. Diabesity Res.,* (this issue).

Forst, T., Kunt, T., Pohlmann, T., Goitom, K., Engelbach, M., Beyer, J., et al. (1998a) Biological activity of C-peptide on skin microcirculation in patients with insulin-dependent diabetes mellitus. *J. Clin. Invest.,* **101,** 2036–2041.

Forst, T., Kunt, T., Pfutzner, A., Beyer, J., and Wahren, J. (1998b) New aspects on biological activity of C-peptide in IDDM patients. *Exp. Clin. Endocrinol. Diabetes,* **106,** 270–276.

Greene, D. A., Lattimer, S. A., and Sima, A. A. F. (1987) Sorbitol, phosphoinositides and sodium-potassium ATPase in the pathogenesis of diabetic complications. *N. Engl. J. Med.,* **316,** 599–606.

Greene, D. A., Sima, A. A. F., Feldman, E. L., and Stevens, M. J. (1997) Diabetic neuropathy. In: *Ellenberg and Rifkin Diabetes Mellitus*, Edited by Rifkin, H., Porte, D., and Sherwin, R., pp. 1009–1076 Stanford, CT, Appleton and Lange.

Greene, D. A., Sima, A. A. F., Stevens, M., Feldman, E., Killen, P., Henry, D., Thomas, T., Dannenberg, J., and Lattimer, S. A. (1993) Aldose reductase inhibitors: An approach to the treatment of the nerve damage of diabetic neuropathy. *Diabetes Metab. Rev.,* **9,** 189–217.

Greene, D. A., Sima, A. A. F., Stevens, M. J., Feldman, E. L., and Lattimer, S. A. (1992) Complications: Neuropathy, pathogenetic considerations. *Diabetes Care,* **15,** 1902–1925.

Grunberger, G., Qiang, X., Li, Z.-G., Mathews, S. T., Sbriessa, D., Shisheva, A., et al. (2001) Molecular basis for the insulinomimetic effects of C-peptide. *Diabetologia,* **44,** 1247–1257.

Grunberger, G., and Sima, A. A. F. (2004) The C-peptide signaling. *J. Diabesity Res.,* **5,** 25–36.

Hammes, H. P., Alt, A., Niwa, T., Clausen, J. T., Bretzel, R. G., Brownlee, M., et al. (1999) Differential accumulation of advanced glycation end products in the course of diabetic retinopathy. *Diabetologia,* **42,** 728–736.

Heesom, A. E., Millward, A., and Demaine, A. G. (1998) Susceptibility to diabetic neuropathy in patients with insulin dependent diabetes mellitus is associated with a polymorphism at the 5′ end of the aldose reductase gene. *J. Neurol. Neurosurg. Psychiatry,* **64,** 213–216.

Hellweg, R., and Hartung, H. D. (1990) Endogenous levels of nerve growth factor (NGF) are altered in experimental diabetes mellitus: A possible role for NGF in the pathogenesis of diabetic neuropathy. *J. Neurosci. Res.,* **26,** 258–267.

Hengerer, B., Lindholm, D., Henmann, R., Ruther, V., Wagner, E. F., and Thoenen, H. (1990) Lesion-induced increase in nerve growth factor mRNA is mediated by c-fos. *Proc. Natl. Acad. Sci. U. S. A.,* **87,** 3899–3903.

Hory-Lee, F., Russell, M., Lindsay, R. M., and Frank, E. (1993) Neurotrophin 3 supports the survival of developing muscle sensory neurons in culture. *Proc. Natl. Acad. Sci. U. S. A.,* **90,** 2613–2617.

Hruska, R. E., Chertak, M. M., and Kravis, D. (1993) Elevation of nerve growth factor receptor—truncated in the urine of patients with diabetic neuropathy. *Ann. N. Y. Acad. Sci.,* **679,** 349–351.

Hunt, J. V., Dean, R. T., and Wolff, S. P. (1988) Hydroxyl radical production and autoxidative glycation. Glucose autoxidation as the cause of protein damage in the experimental glycation model of diabetes mellitus and aging. *Biochem. J.,* **256,** 205–212.

Hunt, J. V., and Wolff, S. P. (1991) Oxidative glycation and free radical production: A causal mechanism of diabetic complications. *Free Radic. Res. Commun.,* **12–13,** 115–123.

Ide, C. (1996) Peripheral nerve regeneration. *Neurosci. Res.,* **25,** 101–121.

Ishii, D. N. (1995) Implication of insulin-like growth factors in the pathogenesis of diabetic neuropathy. *Brain Res. Rev.,* **20,** 47–67.

Ishii, D. N., Guertin, D. M., and Whalen, L. R. (1994) Reduced insulin-like growth factor-I mRNA content in liver, adrenal glands and spinal cord of diabetic rats. *Diabetologia,* **37,** 1073–1081.

Ishii, H., Koya, D., and King, G. L. (1998) Protein kinase C activation and its role in the development of vascular complications in diabetes mellitus. *J. Mol. Med.,* **76,** 21–31.

Ishii, D. N., and Lupien, S. B. (1995) Insulin-like growth factors protect against diabetic neuropathy: Effects on sensory nerve regeneration in rats. *J. Neurosci. Res.,* **40,** 138–144.

Isom, L. L. (2002) The role of sodium channels in cell adhesion. *Front Biosci.,* **7,** 12–23.

Jakobsen, J., Brimijoin, S., Skau, K., Sidenius, P., and Wells, D. (1981) Retrograde axonal transport of transmitter enzymes, fucose-labeled protein, and nerve growth factor in streptozotocin-diabetic rats. *Diabetes,* **30,** 797–803.

Jensen, M. E., and Messina, E. J. (1999) C-peptide induces a concentration-dependent dilation of skeletal muscle arterioles only in presence of insulin. *Am. J. Physiol.,* **276,** H1223–H1228.

Johansson, B.-L., Borg, K., Fernquist-Forbes, E., Kernell, A., Odergren, T., and Wahren, J. (2000) Beneficial effects of C-peptide on incipient nephropathy and neuropathy in patients with type 1 diabetes mellitus. *Diabetes Med.,* **17,** 181–189.

Johansson, B.-L., Kernell, A., Sjöberg, S., and Wahren, J. (1993) Influence of combined C-peptide and insulin administration on renal function and metabolic control in diabetes type 1. *J. Clin. Endocrinol. Metab.,* **77,** 976–981.

Johansson, B.-L., Linde, B., and Wahren, J. (1992) Effects of C-peptide on blood flow, capillary diffusion capacity and glucose utilization in the exercising forearm of type 1 (insulin-dependent) diabetic patients. *Diabetologia,* **35,** 1151–1158.

Kamijo, M., Merry, A. C., Cherian, P. V., Akdas, G., and Sima, A. A. F. (1996) Nerve fiber regeneration following axotomy in the diabetic BB/W-rat. The effect of ARI-treatment. *J. Diabetes Complication,* **10,** 183–191.

Lambert, S., Davis, J. Q., and Bennett, V. (1997) Morphogenesis of the node of Ranvier: Co-clusters of ankyrin and ankyrin-binding integral proteins define early developmental intermediaries. *J. Neurosci.,* **15,** 7025–7036.

Laudadio, C., and Sima, A. A. F. (1996) Design of controlled clinical trials for diabetic polyneuropathy. *Semin. Neurol.,* **16,** 187–192.

Laudadio, C., and Sima, A. A. F., and The Ponalrestat Study Group (1998) Progression rates of diabetic neuropathy in placebo patients in an 18-month clinical trial. *J. Diabetes Complication,* **12,** 121–127.

Li, Z., Zhang, W., Grunberger, G., and Sima, A. A. F. (2002a) Hippocampal neuronal apoptosis in type 1 diabetes. *Brain Res.,* **946,** 212–231.

Li, Z.-G., Qiang, X., Sima, A. A. F., and Grunberger, G. (2001) C-peptide attenuates protein tyrosine phosphatase activity and enhances glycogen synthesis in L6 myoblasts. *Biochem. Biophys. Res. Commun.,* **280,** 615–619.

Li, Z.-G., Zhang, W., and Sima, A. A. F. (2002b) C-peptide prevents hippocampal apoptosis in type 1 diabetes. *Int. J. Exp. Diabetes Res.,* **3,** 241–246.

Li, Z.-G., Zhang, W., and Sima, A. A. F. (2003) C-peptide enhances insulin-mediated cell growth and protects against high glucose induced apoptosis in SH-SY5Y cells. *Diabetes Metab. Res. Rev.,* **19,** 375–385.

Low, P. A., Nickander, K. K., and Scionti, L. (1999) Role of hypoxia, oxidative stress, and excitatory neurotoxins in diabetic neuropathy. In: *Diabetic Neuropathy,* Edited by Dyck, P. J., and Thomas, P. K., pp. 317–329. Philadelphia, W. B. Saunders.

Malhotra, J. D., Kazeu-Gillespie, K., Hortsch, M., and Isom, L. L. (2000) Sodium channel $\beta$ subunits mediate homophilic cell adhesion and recruit ankyrin to points of cell-cell contact. *J. Biol. Chem.,* **275,** 11383–11388.

McCance, D. R., Dyer, D. G., Dunn, J. A., Bailie, K. E., Thorpe, S. R., and Baynes, J. W. (1993) Maillard reaction products and their relation to complications in insulin-dependent diabetes mellitus. *J. Clin. Invest.,* **91,** 2470–2478.

McCord, J. M. (1985) Oxygen-derived free radicals in postischemic tissue injury. *N. Engl. J. Med.,* **312,** 159–163.

Menegoz, M., Gaspar, P., Le Bert, M., Galvez, T., Burgaya, F., Palfrey, C., et al. (1997) Paranodin, a glycoprotein of neuronal paranodal membranes. *Neuron,* **19,** 319–331.

Mitchison, T., and Kirschner, M. (1988) Cytoskeletal dynamics and nerve growth. *Neuron,* **1,** 761–772.

Muller-Husman, G., Gloor, S., and Schachner, M. (1993) Functional characterization of beta isoforms of murine Na/K-ATPase. The adhesion molecule on glia (AMOG/beta 2) but not beta 1, promotes neurite outgrowth. *J. Biol. Chem.,* **268,** 26260–26267.

New, H. V., and Mudge, A. W. (1986) Distribution and ontogeny of SP, CGRP, SOM, and VIP in chick sensory and sympathetic ganglia. *Dev. Biol.,* **116,** 337–346.

Oates, P. J., and Mylari, B. L. (1999) Aldose reductase inhibitors: Therapeutic implications for diabetic complications. *Exp. Opinion Invest. Drugs,* **8,** 2095–2119.

Peles, E., Nativ, M., Campbell, P. L., Sukurai, T., Martinez, R., Lev, S., et al. (1995) The carbonic anhydrase domain of receptor tyrosine phosphatase beta is a functional ligand for the axonal cell recognition molecule contactin. *Cell,* **82,** 251–260.

Peles, E., Nativ, M., Lustig, M., Graumet, M., Schilling, J., Martinez, R., et al. (1997) Identification of a novel contactin-associated transmembrane receptor with multiple domains implicated in protein-protein interactions. *EMBO J.,* **16,** 978–988.

Pierson, C. R., Zhang, W., Murakawa, Y., and Sima, A. A. F. (2002) Early gene responses of trophic factors differ in nerve regeneration in type 1 and type 2 diabetic neuropathy. *J. Neuropathol. Exp. Neurol.,* **61,** 857–871.

Pierson, C. R., Zhang, W., Murakawa, Y., and Sima, A. A. F. (2003a) Insulin deficiency rather than hyperglycemia accounts for impaired neurotrophic responses and nerve fiber regeneration in type 1 diabetic neuropathy. *J. Neuropathol. Exp. Neurol.,* **63,** 260–271.

Pierson, C. R., Zhang, W., and Sima, A. A. F. (2003b) Proinsulin C-peptide replacement in type 1 BB/Wor-rats prevents deficits in nerve fiber regeneration . *J. Neuropathol. Exp. Neurol.,* **62,** 765–779.

Pirart, J. (1977) Diabetes mellitus and its degenerative complications: A prospective study of 4,400 patients observed between 1947 and 1973. *Diabete Metab.,* **3,** 97–107.

Ratcliff, C. F., Qu, Y., McCormick, K. A., Tibbs, V. C., Dixon, J. E., Scheuer, T., et al. (2000) A sodium channel signaling complex: Modulation by associated receptor protein tyrosine phosphatase $\beta$. *Nat. Neurosci.,* **3,** 437–444.

Rigler, R., Pramanik, A., Jonasson, P., Kratz, G., Jansson, O. T., Nygren, P., et al. (1999) Specific binding of proinsulin C-peptide to human cell membranes. *Proc. Natl. Acad. Sci. U. S. A.,* **96,** 13318–13323.

Ryle, C., Leow, C. K., and Donaghy, M. (1997) Nonenzymatic glycation of peripheral and central nervous system proteins in experimental diabetes mellitus. Muscle Nerve, **20,** 577–584.

Scherer, S. S. (1996) Molecular specialization at nodes and paranodes in peripheral nerve. *Micros. Res. Techn.,* **34,** 452–461.

Sell, D. R., Lapolla, A., Odetti, P., Fogarty, J., and Monnier, V. M. (1992) Pentosidine formation in skin correlates with severity of complications in individuals with long-standing IDDM. *Diabetes,* **41,** 1286–1292.

Sima, A. A. F. (1994) Pathological definition and evaluation of diabetic neuropathy and clinical correlations. *Can. J. Neurol. Sci.,* **21(Suppl 4),** 513–517.

Sima, A. A. F. (1996) Metabolic alterations of peripheral nerve in diabetes. *Semin. Neurol.,* **16,** 129–137.

Sima, A. A. F. (1999) Diabetic neuropathy—The utility of nerve biopsy, In: *Clinical Neurophysiology: From Receptors to Perception*, Edited by Comi, G., Lücking, C. H., Kimura, J., Rossini, P. M., pp. 525–533. Amsterdam, Elsevier Science.

Sima, A. A. F. (2001) Diabetic neuropathy; pathogenetic backgrounds, current and future therapies. *Expert Rev. Neurother.,* **1,** 225–238.

Sima, A. A. F. (2003a) New insights into the metabolic and molecular basis for diabetic neuropathy. *Cell. Mol. Life Sci.,* **60,** 1–20.

Sima, A. A. F. (2003b) C-peptide and diabetic neuropathy. *Expert Opin. Drug Dev.,* **12,** 1471–1488.

Sima, A. A. F., Bril, V., Nathaniel, V., McEwen, T. A. J., Brown, M., Lattimer, S. A., and Greene, D. A. (1988a) Regeneration and repair of myelinated fibers in sural nerve biopsies from patients with diabetic neuropathy treated with an aldose reductase inhibitor. *N. Engl. J. Med.,* **319,** 548–555.

Sima, A. A. F., and Brismar, T. (1985) Reversible diabetic nerve dysfunction. Structural correlates to electrophysiological abnormalities. *Ann. Neurol.,* **18,** 21–29.

Sima, A. A. F., and Cherian, P. V. (1997) Neuropathology of diabetic neuropathy and its correlations with neurophysiology. *Clin. Neurosci.,* **4,** 359–364.

Sima, A. A. F., Grunberger, G., Jörnvall, H., Wahren, J., and The C-Peptide Study Group (2001a) Proinsulin C-peptide—A consensus statement. *Int. J. Exp. Diabetes Res.,* **2,** 145–151.

Sima, A. A. F., Lattimer, S. A., Yagihashi, S., and Greene, D. A. (1986) "Axo-glial dysjunction": A novel structural lesion that accounts for poorly reversible slowing of nerve conduction in the spontaneously diabetic BB-rat. *J. Clin. Invest.,* **77,** 474–484.

Sima, A. A. F., Li, Z., Zhang, W., and Stevens, M. (2003a) Impaired endoneurial blood flow but not oxidative stress is prevented by C-peptide in type 1 BB/Wor-rats. *Brain Pathol.,* **12(Suppl 41)**.

Sima, A. A. F., Merry, A. C., and Lightle, R. (1997a) Impaired macrophage recruitment in axotomized diabetic nerve. *Exp. Clin. Endocrinol Diabetes.,* **105,** 62–63.

Sima, A. A. F., Nathaniel, V., Bril, V., McEwen, T. A. J., and Greene, D. A. (1988b) Histopathological heterogeneity of neuropathy in insulin-dependent and non-insulin-dependent diabetes, and demonstration of axo-glial dysjunction in human diabetic neuropathy. *J. Clin. Invest.,* **81,** 349–364.

Sima, A. A. F., Pierson, C. R., and Zhang, W. (2002) C-peptide prevents the molecular abnormalities of the paranode in type 1 diabetic polyneuropathy (DPN). Presented at the 18th International Diabetes Federation Congress, Paris, abstract.

Sima, A. A. F., Pierson, C. R., and Zhang, W. (2003b) The molecular abnormalities of the paranode in type 1 diabetic polyneuropathy are prevented by C-peptide. *Brain Pathol.* **12(Suppl. 42),** 17, abstract.

Sima, A. A. F., and Sugimoto, K. (1999) Experimental diabetic neuropathy: An update. *Diabetologia,* **42,** 773–788.

Sima, A. A. F., Thomas, P. K., Ishii, D., and Vinik, A. (1997b) Diabetic neuropathies. *Diabetologia,* **40(Suppl 3),** B74–B77.

Sima, A. A. F., Zhang, W., Xu, G., Sugimoto, K., Guberski, D. L., and Yorek, M. A. (2000) A comparison of diabetic polyneuropathy in type-2 diabetic BBZDR/Wor-rat and in type 1 diabetic BB/Wor-rat. *Diabetologia,* **43,** 786–793.

Sima, A. A. F., Zhang, W.-X., Sugimoto, K., Henry, D., Li, Z.-G., Wahren, J., et al. (2001b) C-peptide prevents and improves chronic type 1 diabetic neuropathy in the BB/Wor-rat. *Diabetologia,* **44,** 889–897.

Soulis, T., Thallas, V., Youssef, S., Gilbert, R. E., McWilliam, B. G., Murray-McIntosh, R. P., et al. (1997) Advanced glycation end products and their receptors co-localise in rat organs susceptible to diabetic microvascular injury. *Diabetologia,* **40,** 619–628.

Stevens, M. J., Dananberg, J., Feldman, E. L., Lattimer, S. A., Kamijo, M., Thomas, T. P., Shindo, H., Sima, A. A. F., and Greene, D. A. (1994) The linked roles of nitric oxide, aldose reductase and, $(Na^{+}/K^{+})$-ATPase in the slowing of nerve conduction in the streptozotocin diabetic rat. *J. Clin. Invest.,* **94,** 853–859.

Stevens, M. J., Lattimer, S. A., Kamijo, M., Van Huysen, C., Sima, A. A. F., and Greene, D. A. (1993) Osmotically-induced nerve taurine depletion and the compatible osmolyte hypothesis in experimental diabetic neuropathy in the rat. *Diabetologia,* **36,** 608–614.

Stevens, M., Li, F., Zhang, W., and Sima, A. A. F. (2003) C-peptide prevents impaired endoneurial blood flow but does not effect oxidative stress in type 1 BB/Wor-rats. *J. Periph. New Syst.,* **8,** 196.

Sugimoto, K., Murakawa, Y., and Sima, A. A. F. (2000a) Diabetic neuropathy—a continuing enigma. *Diabetes Metab. Res. Rev.,* **16,** 408–433.

Sugimoto, K., Murakawa, Y., Zhang, W.-X., Xu, G., and Sima, A. A. F. (2000b) Insulin receptor in rat peripheral nerve: Its localization and alternatively spliced isoforms. *Diabetes Metab. Res. Rev.,* **16,** 354–363.

Tesfaye, S., Stevens, L. K., Stephanson, J. M., Fuller, J. H., Plater, M., Ionescu-Tirgoviste, C., et al. (1996) Prevalence of diabetic peripheral neuropathy and its relation to glycaemic control and potential risk factors: The EURODIAB IDDM Complication Study. *Diabetologia,* **39,** 1377–1384.

Thomas, P. K. (1997) Classification, differential diagnosis, and staging of diabetic peripheral neuropathy. *Diabetes,* **46(Suppl 2),** S54–S57.

Thomas, P. K., and Eliasson, S. G. (1984) Diabetic neuropathy. In: *Peripheral Neuropathy*, Edited by Dyck, P. J., Thomas, P. K., Lambert, E. H., and Bunger, R., pp. 1973–1810. Philadelphia, W. B. Saunders.

Tomlinson, D. R., and Fernyhough, P. (1999) Neurotrophism in diabetic neuropathy. In: *Chronic Complications in Diabetes. Animal Models and Chronic Complications,* Edited by Sima, A. A. F., pp. 167–182. Amsterdam, Harwood.

Tomlinson, D. R., Fernyhough, P., and Diemel, L. T. (1997) Role of neurotrophins in diabetic neuropathy and treatment with nerve growth factors. *Diabetes,* **46(Suppl 2),** S43–S49.

Vinik, A. I., Liuzze, F. J., Holland, M. T., Stansberry, K. B., Lebean, J. M., and Colen, L. B. (1992) Diabetic neuropathies. *Diabetes Care,* **15,** 1926–1975.

Vlassara, H., Brownlee, M., and Cerami, A. (1981) Nonenzymatic glycosylation of peripheral nerve protein in diabetes mellitus. *Proc. Natl. Acad. Sci. U. S. A.,* **78,** 5190–5192.

Vlassara, H., Brownlee, M., and Cerami, A. (1984) Accumulation of diabetic rat peripheral nerve myelin by macrophages increases with the presence of advanced glycosylation endproducts. *J. Exp. Med.,* **160,** 197–207.

Vlassara, H., Brownlee, M., and Cerami, A. (1985) Recognition and uptake of human diabetic peripheral nerve myelin by macrophages. *Diabetes,* **34,** 553–557.

Wahren, J., Ekberg, K., Johansson, J., Henriksson, M., Pramanik, A., Johansson, B.-L., et al. (2000) Role of C-peptide in human physiology. *Am. J. Physiol. Endocrinol. Metab.,* **278,** E759–E768.

Wallerath, T., Kunt, T., Forst, T., Closs, E., Lehmann, R., Göpfert, A., Pfütgner, A., Beyer, J., and Förstermann, U. (2003) Stimulation of endothelial nitric oxide synthase by proinsulin C-peptide. *Am. J. Physiol.,* in press.

Wautier, J. L., Zoukourian, C., Chappey, O., Wautier, M. P., Guillausseau, P. J., Cao, R., et al. (1996) Receptor-mediated endothelial cell dysfunction in diabetic vasculopathy. Soluble receptor for advanced glycation end products blocks hyperpermeability in diabetic rats. *J. Clin. Invest.,* **97,** 238–243.

Williamson, J. R., Chang, K., Frangos, M., Hasan, K. S., Ido, Y., Kawamura, T., et al. (1993) Hyperglycemic pseudohypoxia and diabetic complications. *Diabetes,* **42,** 801–813.

Wuarin, L., Guertin, D. M., and Ishii, D. N. (1994) Early reduction in insulin-like growth factor gene expression in diabetic nerve. *Exp. Neurol.,* **130,** 106–114.

Xu, G., Murakawa, Y., Pierson, C. R., and Sima, A. A. F. (2002) Altered ß-tubulin and neurofilament expression and impaired axonal growth in diabetic nerve regeneration. *J. Neuropathol. Exp. Neurol.,* **61,** 164–175.

Xu, G., and Sima, A. A. F. (2001) Altered immediate early gene expression is impaired in diabetic nerve: Implications in regeneration. *J. Neuropathol. Exp. Neurol.,* **60,** 972–983.

Yagihashi, S., Kamijo, M., and Watanabe, K. (1990) Reduced myelinated fiber size correlates with loss of axonal neurofilaments in peripheral nerve of chronically streptozotocin diabetic rats. *Am. J. Pathol.,* **136,** 1365–1373.

Yoshida, K., Hirokawa, J., Tagami, S., Kawakami, Y., Urata, Y., and Kondo, T. (1995) Weakened cellular scavenging activity against oxidative stress in diabetes mellitus: Regulation of glutathione synthesis and efflux. *Diabetologia,* **38,** 201–210.

Zhang, W., Yorek, M., Pierson, C. R., Murakawa, Y., Breidenbach, A., and Sima, A. A. F. (2001) Human C-peptide dose dependently prevents early neuropathy in the BB/Wor-rat. *Int. J. Exp. Diabetes Res.,* **2,** 187–194.

Zhu, X., and Eichberg, J. (1990) A *myo*-inositol pool utilized for phosphatidylinositol synthesis is depleted in sciatic nerve from rats with streptozotocin-induced diabetes. *Proc. Natl. Acad. Sci. U. S. A.,* **87,** 9818–9822.

Zhuang, H. X., Snyder, C. K., Pu, S. F., and Ishii, D. N. (1996) Insulin-like growth factors reverse or arrest diabetic neuropathy: Effects on hyperalgesia and impaired nerve regeneration in rats. *Exp. Neurol.,* **140,** 198–205.

Zhuang, H. X., Wuarin, L., Fei, Z. J., and Ishii, D. N. (1997) Insulin-like growth factor (IGF) gene expression is reduced in neural tissues and liver from rats with non-insulin-dependent diabetes mellitus, and IGF treatment ameliorates diabetic neuropathy. *J. Pharmacol. Exp. Ther.,* **283,** 366–374.

Ziegler, D., Reljanovic, M., Meknert, H., and Gries, F. A. (1999) Alpha lipoic acid in the treatment of diabetic polyneuropathy in Germany: Current evidence from clinical trials. *Exp. Clin. Endocrinol. Diabetes,* **107,** 421–430.

